# Biosynthesis of Copper Oxide (CuO) Nanowires and Their Use for the Electrochemical Sensing of Dopamine

**DOI:** 10.3390/nano8100823

**Published:** 2018-10-12

**Authors:** Sasikala Sundar, Ganesh Venkatachalam, Seong Jung Kwon

**Affiliations:** 1 Department of Chemistry, Konkuk University, 120 Neungdong-ro, Gwangjin-gu, Seoul 143-701, Korea; sasikala412@gmail.com; 2 Electrodics and Electrocatalysis (EEC) Division, CSIR–Central Electrochemical Research Institute (CSIR–CECRI), Karaikudi 630003, Tamilnadu, India

**Keywords:** biosynthesis, copper oxide, nanowires, dopamine, electrochemical biosensor

## Abstract

A facile one-step, eco-friendly, and cost-effective approach for the formation of copper oxide (CuO) nanowires by a green method using saponin-rich *Sapindus mukorossi* fruit extract (SMFE). The physio-chemical characteristics of the synthesized CuO nanowires have been characterized by X-ray Diffractometry (XRD), X-ray Photoelectron Spectroscopy (XPS), FT-IR (Fourier Transform Infrared Spectroscopy, FE-SEM (Scanning Electron Microscopy), and High-Resolution Transmission Electron Microscopy (HR-TEM). Further, the electrocatalytic activity of the CuO nanowires synthesized with SMFE has been investigated, and they have been used as dopamine (DA) sensors. Because of their unique properties, the CuO nanowires/GCE exhibited remarkable electrochemical response for the detection of DA with enhanced current response. The anodic current demonstrated that the CuO nanowires/GCE linearly detects the concentration of DA over the range of 0.1 µM to 0.105 mM of DA with a regression co-efficient of 0.9960. The obtained results illustrated that the synthesized CuO nanowires can easily stimulate the electron transfer reaction between DA and the nanowires modified electrode with the improvement of the conductivity and stability of the electrode. This remarkable electrocatalytic property of CuO nanowires makes it a unique electrochemical sensor for the detection of DA. Furthermore, the sensor is free from the interference of ascorbic acid, uric acid, and other interfering species. Moreover, the anti-interference performance also showed that the CuO nanowires/GCE could be employed for the determination of DA in real samples with good selectivity and sensitivity.

## 1. Introduction

Owing to the attractive physicochemical properties of transition metal or metal oxide nanostructured materials, significant attempts have been promoted towards the preparation of such metal/metal oxide nanoparticles with desired shapes and sizes [1,2]. Among the transition metal oxides, CuO is one of the best known naturally occurring *p*-type semiconductors, and in particular, nanostructured copper oxides are especially versatile and they offer unique characteristics in many applications in sensors, high-critical-temperature superconductors, lithium-ion batteries, field emission emitters, and catalysts [3,4,5,6,7]. The size and morphology of the CuO nanostructured materials considerably affect their electrochemical activity [8]; therefore, controlling the morphology and the enhancement of the cyclability of CuO-based electrodes are crucial. In order to improve these novel properties, synthesis of CuO nanostructures in the form of nanowires, nanorods, nanoneedles, nanoflowers, and nanoparticles, which show excellent electrochemical performance as compared to their bulk counterparts. Over the past few years, variety of preparation methods have been developed to generate CuO nanoparticles with varying dimensions and structures such as the self-catalytic mechanism, simple solution preparation, hydrolysis, hydrothermal and solvothermal synthesis, and exposure to microwave irradiation [9,10,11,12,13,14]. However, these chemical methods involve the use of toxic, very expensive, hazardous, and non-environmentally friendly chemicals, thus they are not acceptable for the various fields of biomedical applications. Hence, the influence of green synthesis provides advancement over chemical and physical method, as it is cost effective, eco-friendly, easily scaled up for large scale synthesis, and provides no need to use high pressure, energy, temperature, and toxic chemicals for the synthesis of morphology controlled nanostructured materials, which will find extensive use in biomedicine [15].

Several types of neurotransmitter related diseases, such as Parkinson’s and Alzheimer’s diseases, epilepsy and schizophrenia, can be caused by the abnormal metabolism and concentration of Dopamine (4-(2-aminoethyl)benzene-1,2-diol) [16]. For this reason, and because of its important role in physiology and pathophysiology, it is necessary to explore a cost effective, sensitive and reliable quantitative method for the determination of dopamine (DA) in the biological fluids. Compared with traditional analytical methods, electroanalytical methods are highly sensitive and selective, rapid and inexpensive [17]. When compared to the metal electrodes, nanostructured metal oxide fabricated electrode materials provide some unique electrochemical characteristics and induces higher reaction rates with the exclusion of weakly adsorbed hydrogen species being present on the surface of the oxides [18]. Various types of nanostructured metal oxides, like ZnO nanowires, RGO-TiO_2_ nanocomposites, RuO_2_/CNT, Fe_3_O_4_ nanorods/grapheme, Fe_3_O_4_@Au nanoparticles, graphene/SnO_2_, and WO_3_ nanoparticles, have been efficiently developed for the determination of DA in the biological samples [19,20,21,22,23,24,25]. Among these electrocatalysts, distinctive properties of copper oxide (CuO) have achieved higher interest, especially in the fields of electrochemical biosensors [26,27,28,29,30]. Based on the previous investigations and experience on the synthesis of different phases of magnetic (iron oxides) metal oxides, such as Fe_3_O_4_, γ-Fe_2_O_3_, and α-Fe_2_O_3_ using *Sapindus mukorossi* fruit extract (SMFE) [31] for the development of electrochemical biosensors, we have examined here the electrocatalytic behavior of one-dimensional copper oxide nanowires modified GCE synthesized using SMFE for the sensitive and selective determination of DA.

## 2. Materials and Methods

### 2.1. Materials

Copper (II) sulfate hexahydrate (CuSO_4_·6H_2_O), sodium hydroxide (NaOH) were purchased from Merck (Mumbai, India). *Sapindus mukorossi* fruits were obtained from the local market (Tamil Nadu, India). Uric acid (UA), ascorbic acid (AA), citric acid (CA), dopamine (DA), glucose, sodium chloride (NaCl), dihydrogen orthophosphate and dipotassium hydrogen phosphate were obtained from Sigma-Aldrich (St. Louis, MO, USA). All of the above purchased chemicals were used without any additional purification process. Ultrapure water (>18 MΩ, Millipore, Darmstadt, Germany) was used in all the experiments.

### 2.2. Preparation of Sapindus Mukorossi Fruit Extract (SMFE)

About 10 g of the dried fruits were dissolved in conical flask containing 100 mL water and stirred for 12 h. The brownish yellow colored extracted solution was filtered through Whatman No. 1 filter paper. Finally, the collected filtrate was kept at 4 °C and was used for the further experimental process.

### 2.3. Preparation of CuO Nanowires Using SMFE

Typically, CuO nanowires were prepared by a simple precipitation process that was based on a modified version of the procedure reported in the literature [31]. A stoichiometric amount of 0.1 M CuSO_4_·6H_2_O was mixed with 2 mL of SMFE and stirred well for 10 min. An aqueous solution of 0.1 M NaOH was introduced slowly into the above mixture until it reaches pH 11 and allowed to stir for 2 h at ambient atmosphere. The obtained bluish green precipitate indicates the formation of Copper (II) hydroxide, and then the resultant precipitate was centrifuged and washed for 2–3 times using de-ionized water. Finally, the decanted precipitate was dried in an oven for 24 h at 80 °C and placed in a preheated furnace at 300 °C for 1 h to get the CuO nanostructures. The above-mentioned synthesis method was performed in the greener environment without the presence of any inert gases or a vacuum atmosphere.

### 2.4. Characterizations of CuO Nanowires

The green synthesized copper oxide nanowires were characterized by various physiochemical techniques. X-ray diffractometry (XRD) was recorded to identify the crystal structure with CuKα radiation (PANalytical X’Pert Pro Diffractometer, Almelo, The Netherlands). X-ray Photoelectron Spectrometer (XPS) of the CuO nanowires was used to confirm the chemical compositions and the oxidation states of the formed products by Kratos ASIS-HS, X-ray photoelectron spectrometer (Thermo Scientific equipment, Warrington, UK). Fourier Transform Infrared Spectroscopy (FTIR) was recorded to examine the chemical binding nature of the synthesized products using a Bruker IR spectrometer (Bruker Optics, Victoria, Australia). The morphology and size of the CuO nanowires were characterized while using a Field Emission-Scanning Electron Microscopy (FE-SEM, Hitachi model S3000-H), Hitachi Science & Technology, UK and High Resolution-Transmission electron microscopy (HR-TEM, TECNAI G^2^ 20), FEI Company, Hillsboro, OR, USA.

## 3. Results and Discussion

### 3.1. XRD Patterns of the CuO Nanowires

**Figure 1:**
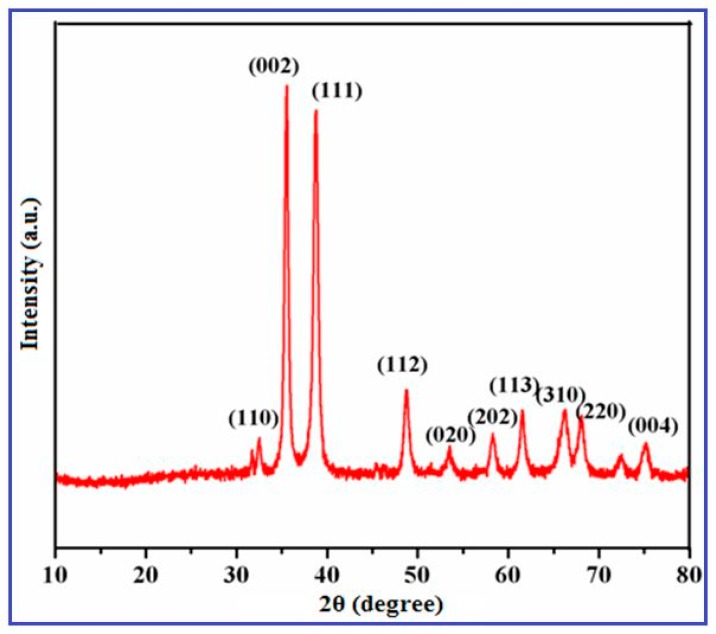
X-ray diffractometry (XRD) pattern of copper oxide (CuO) nanowires synthesized using SMFE.

The phase purity and structural characteristics of the synthesized CuO nanowires were carried out using XRD analysis, which are shown in the Figure 1. The XRD Diffraction patterns appeared at 2θ (32.45°, 35.57°, 38.69°, 48.77°, 53.57°, 58.75°, 61.72°, 66.04°, 68.24° and 75.16°) values and they were assigned to the corresponding (110), (002), (111), (112), (020), (202), (113), (310), (220) and (004) planes, respectively.

The obtained XRD patterns indicated that the synthesized nanowires are highly crystalline with monoclinic structure of CuO, which was confirmed by the Joint Committee on Powder Diffraction Standards (JCPDS) (Card No.: 89-5895). The other impurity products like Cu(OH)_2_ and Cu_2_O characteristic peaks were not observed, revealing the phase pure formation of CuO nanowires [32]. The peak broadening also noticeably signifies the development of smaller sized CuO nanowires. The average crystallite size of the CuO particles that were synthesized by this greener approach was estimated to be ca. 46.2 nm by using the Debye–Scherrer formula, *D* = *K*λ/*β*cos*θ.* These diffraction patterns appeared in this study are similar to those previously reported literatures about the eco-friendly preparation of CuO nanoparticles [33,34]. The XRD results further indicate that the CuO nanowires synthesized through this biosynthesis method constitute single-phase growth with high degree of purity and confirmed the existence of CuO.

### 3.2. XPS Spectra of the CuO Nanowires

**Figure 2:**
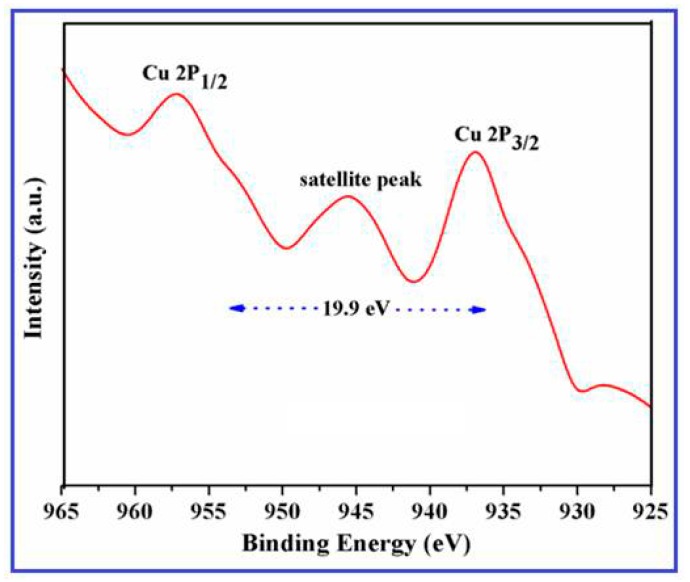
Core level X-ray Photoelectron Spectrometer (XPS) spectral peaks of CuO nanowires synthesized using *Sapindus mukorossi* fruit extract (SMFE).

XPS spectra were performed to analyze the chemical states of transition metals, including their localized valence d-orbitals. Figure 2 illustrates the core level Cu 2p high-resolution photoemission spectra of the CuO nanowires synthesized using SMFE. As shown in the XPS spectra, the binding energy emerged at around 936.5 eV and 956.4 eV are corresponding to the core level peaks of Cu 2p_3/2_ and Cu 2p_1/2_ that are present in the pure phase of CuO nanowires.

The emergence of strong satellite peak and the binding energy gap value (19.9 eV) between the Cu 2p_3/2_ and Cu 2p_1/2_ peaks is differentiated from the pure Cu or Cu_2_O, which also explores the existence of an unfilled Cu3d^9^ shell, further confirming the presence of Cu^2+^ ions in the CuO [35,36]. The XPS results are also very well matched with the XRD pattern in Figure 1, and they confirm that the synthesized nanowires comprising a pure phase of CuO.

### 3.3. FTIR Spectra of the CuO Nanowires

**Figure 3:**
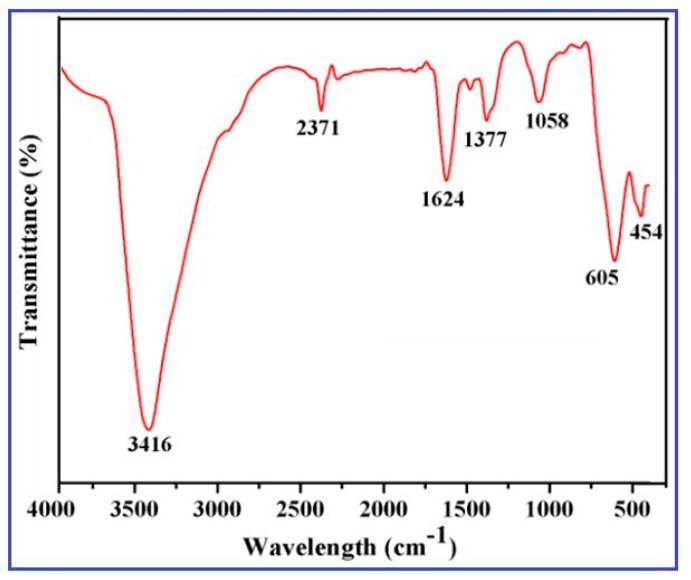
Fourier Transform Infrared Spectroscopy (FTIR) spectra of CuO nanowires synthesized using SMFE.

The CuO nanowires were subjected to FTIR analysis at room temperature to evaluate the chemical composition and to confirm the formation of CuO, which are recorded in the range of 400–4000 cm^−1^. FTIR spectra of CuO nanowires synthesized using SMFE are shown in Figure 3.

The two important ant characteristic bands appeared at around 454.5 cm^−1^ and 605.7 cm^−1^ can be assigned to the A_u_ and B_u_ modes of CuO, respectively [37]. These high-frequency modes of A_u_ and B_u_ can be assigned to the Cu–O stretching vibration along the [1 0 1] direction [38]. An intense broad band appeared in the 3200–3550 cm^−1^ region that was attributed to the O–H stretching vibration of surface hydroxyl groups of adsorbed water molecules [39], which arises because nanocrystalline materials having a high surface-to-volume ratio absorbs high moisture. The small band at approximately 2371 cm^−1^ is due to O=C=O stretching vibration. The sharp absorption band appeared at around 1624 cm^−1^ can be ascribed to the C=C aromatic bending vibration of alkenes present in the SMFE [40] and also implies the formation of bidentate ligand coordination expected between C–O and Cu(II) of CuO. The IR band that appeared at around 1058 cm^−1^ can be attributed to C–OH stretching and OH bending vibration, indicating the existence of a greater number of hydroxyl groups in the chemical structure of SMFE. The IR bands observed in the above-mentioned regions confirmed the formation of CuO in the nanophase, which is also reported by the several researchers [41]. The transmittance peak appeared at around 1377 cm^−1^ may be ascribed due to the presence of CO_2_, which is usually adsorbed from the air on the surface of sample materials during KBr pelletization.

### 3.4. FE-SEM Pictures of the CuO Nanowires Synthesized Using SMFE

**Figure 4:**
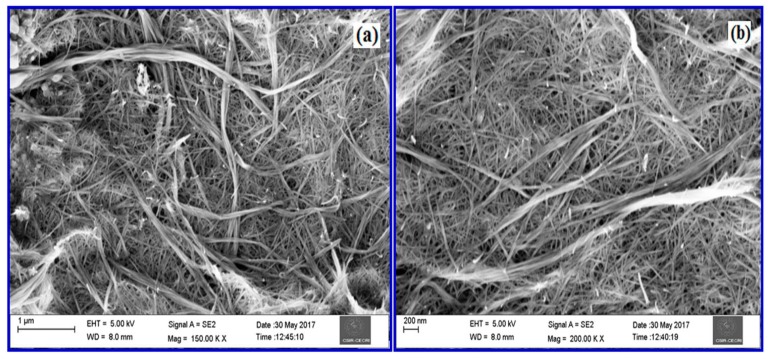
Field Emission-Scanning Electron Microscopy (FE-SEM) micrographs of CuO nanowires prepared using SMFE (**a**) low magnification (150.00 KX) and (**b**) high magnification (200.00 KX).

FE-SEM analysis was performed to investigate the surface morphology and dimension of the as-prepared CuO nanowires. The FE-SEM images that were taken at low and high magnification for the CuO nanowires are shown in Figure 4a,b.

From the observed images, it can be inferred that the CuO nanostructures possess a variable-dimension wire-like morphology. It can also be demonstrated that the final products consist of large number of CuO nanowires of approximately 800 nm in length and 50–100 nm in width that are made up of several small nanowires with a width of about 10 nm, forming a uniform one-dimensional (1D) nanostructure. Clearly, CuO nanowires could be produced in large quantities using this facile method.

### 3.5. HR-TEM Pictures of the CuO Nanowires Synthesized Using SMFE

**Figure 5:**
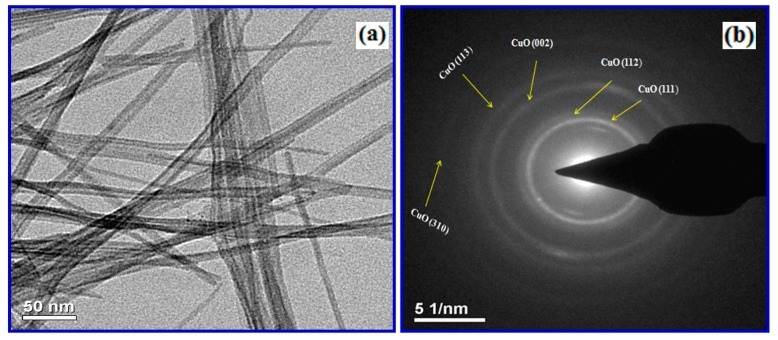
(**a**) High Resolution-Transmission electron microscopy (HR-TEM) micrographs of CuO nanowires synthesized using SMFE and (**b**) selected area electron diffraction (SAED) pattern of corresponding micrograph.

We have examined the microstructure and crystallinity of the CuO nanowires that were grown on the copper grids by HR-TEM and selected area electron diffraction (SAED). Figure 5a,b shows typical HR-TEM and SAED images of discrete CuO nanowires, respectively.

A wire-like morphology was observed for the CuO nanostructures that were synthesized using SMFE, which is given in the Figure 5a and the observed image is also agreed well with the FE-SEM images shown in the Figure 4a,b. Figure 5a illustrated that the diameters of as-obtained nanowires are uniform along the length of nanowires. The main parts of the nanowires are straight, lacking any perceptible tapering at the ends and without any clustering at the tip with a smooth morphology. Figure 5b shows the SAED patterns that were recorded when the electron beam was focused on an individual nanowire shown in Figure 5a. The ring pattern indicates that these CuO nanowires are randomly oriented, and the strong intensities reveal the high crystallinity of the sample. All of the obtained rings in SAED may be assigned to the diffraction peaks of monoclinic structure of CuO rather than those of Cu_2_O and Cu, indicating that the obtained nanowires are in the pure phase of CuO. Furthermore, the indexed diffraction rings can be attributed to the five major crystal planes, (111), (112), (002), (113) and (310), of monoclinic CuO, corroborating with the XRD results [42].

### 3.6. Growth Mechanisms of CuO Nanowires

**Scheme 1:**
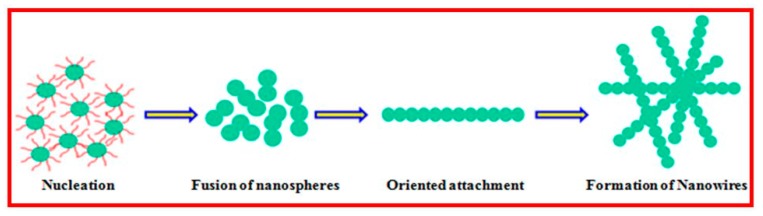
A possible mechanism for the growth of nanospheres into nanowire.

Anisotropic nanostructures, such as nanoribbons, nanowires, nanotubes, nanobelts, nanoleaves, nanorods and nanorods, etc., have attracted immense interest in various applications, because of their unique electronic, chemical, optical and electrocatalytic properties. Therefore, it is highly desirable to synthesize morphology controlled nanostructured materials by various preparation methods. However, such procedures are lengthy and difficult to accomplish with good control over the crystallinity and phase purity of the nanomaterials. To alleviate the above discussed limitations, for the first time the SMFE (bio-surfactant) was employed to synthesize the controlled and directed growth of CuO nanowires, like morphology through greener approach. In addition, SMFE played a though provoking role by generating a large number of well-defined CuO nanowires have been fabricated by the template free bio-surfactant assisted method, and the suggested formation mechanism of 1D nanowire like morphology is illustrated in Scheme 1.

**Figure 6:**
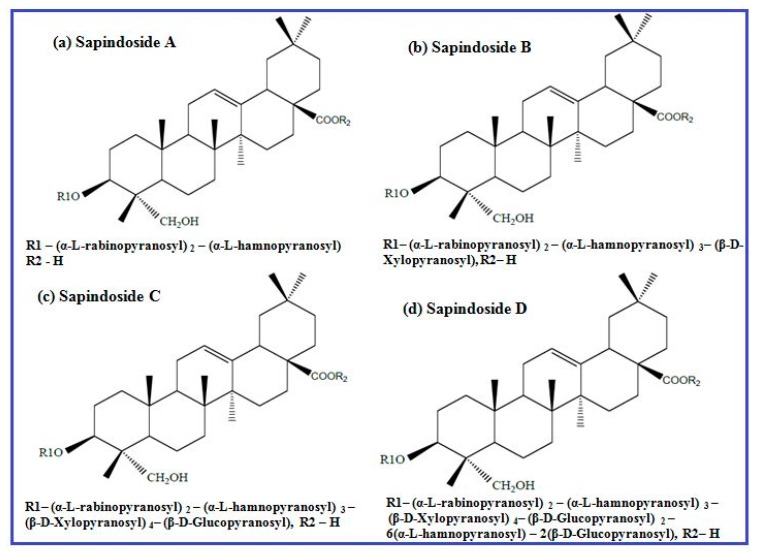
Chemical Structure of different types of saponin present in the SMFE.

The 1D nanostructure formation is an integrated process of nucleation, coalescence, oriented attachment and growth. The aqueous extract of SMFE mainly contains saponin rich compounds, like sapindoside A, sapindoside B, sapindoside C and sapindoside D, as well as *Mukorossi* saponins E1 and Y1, which have many –OH groups, and these compounds act as structure-directing agents, and induces copper hydroxide nanowires, like morphology, some of the chemical structures of saponin present in the SMFE is given in the Figure 6 [31]. Subsequently, the biosurfactant molecules present in the SMFE act as a shape inducing agent for the evolution process of the CuO nanowires, as seen from the FE-SEM and TEM images (Figure 4 and Figure 5).

In an aqueous solution, a simple chemical reaction takes place between precursor (CuSO_4_·6H_2_O) and the alkali (NaOH) by the precipitation of the intermediate product Copper(II) hydroxide (Cu(OH)_2_), acts as a building unit for the tailoring of CuO nanocrytals. It may be assumed that, at an earlier period of reaction time, Cu(OH)_2_ units are produced by the addition of a smaller amount of NaOH, however it is insufficient to produce OH^−^ ions. By exploiting the SMFE having big polar head and long hydrocarbon chain with large number of hydroxyl (–OH) groups, thus facilitates the building up of the nano-architecture of copper oxide with different shapes. Moreover, the slow hydrolysis of transition metal salts helps the organic moiety to control the branching of building blocks lead to a guided 1D growth. Thus, during the formation process of Cu(OH)_2_ units along with the SMFE, it is evident to generate larger amount of complex anionic product Cu(OH)_4_^2−^. It is also believed that a large amount of (Cu(OH)_2_) nanospheres might be produced by the dehydration of [Cu(OH)_4_]^2−^ ions and it serves as a nucleating embryo for further growth of nanowire formation through oriented attachment mechanism. The involvement of higher amount of hydroxyl groups induced by the big polar hydrophilc head and long chain hydrocarbon tail of saponin compounds in SMFE can markedly promote the growth of 1D nanowires formation from the initially developed (Cu(OH)_2_) nanodots/nuclei [43]. It seems that the participation of the bio-surfactant not only renders the [Cu(OH)_4_]^2−^ ions, but also accelerates the 1D nanostructure in a particular direction [44]. Moreover, the concentration of hydroxyl groups are essential for the oriented attachment of (Cu(OH)_2_) nanodots into a linear chain through a crystallographic orientation [45]. Finally, at the calcinations process of Cu(OH)_2_ nanowires, thermal decomposition occurs by forming CuO nanowires like structure. From these observations, it is concluded that the high surface energy of the initially formed nanodots linearly attached through particular planes is driven by the existence of organic molecules in the SMFE, and the dipolar attraction originates from the opposite polarity of the crystal planes (<002> and <111>) directed towards the CuO nanowires.

### 3.7. Electrochemical Investigations of the CuO Nanowires

All of the electrochemical measurements were recorded using a CHI model 660 potentiostat (CH Instruments, Austin, TX, USA) equipped with a common three-electrode assembly, including glassy carbon electrode (GCE) as the working electrode, a platinum wire and saturated calomel (Ag/AgCl) electrodes were used as the counter and reference electrode, respectively. Prior to the modification, the GCE electrode was polished with different grades (0.3 μm and 1 μm) of alumina slurry and ultrasonically rinsed with water and ethanol for the removal of adsorbed impurities. About 4 mg of CuO nanoparticles were suspended in a mixture of 1 mL water and 0.2 mL nafion undergoes sonication for 10 mins until the appearance of syrupy black colored solution. CuO nanowires suspension (10 μL) of the dispersed sample was drop casted on the surface of the pre-cleaned GCE and it serves as a working electrode. The cyclic voltammetry (CV) measurements were carried out from −0.2 V to 0.4 V (vs. Ag/AgCl) at a scan rate of 50 mV·s^−1^ in 0.1 M PBS, pH 7.4. The differential pulse voltammetry (DPV) studies were performed from the potential range −0.2 V to 0.4 V (vs. Ag/AgCl), at a preset pulse width of 0.05, pulse period of 0.5 s, and pulse amplitude of 0.05 V. DPV technique was also used in the multipotential modality to indentify the other interfering biomolecules. Chronoamperometry (CA) study of the modified GCE with the consecutive addition of the common interfering species was carried in 0.1 M phosphate buffer (pH 7.4) at a given potential of 0.15 V (vs. Ag/AgCl).

### 3.8. Electrochemical Sensing Behavior of the CuO Nanowire/GCE for Dopamine

**Figure 7:**
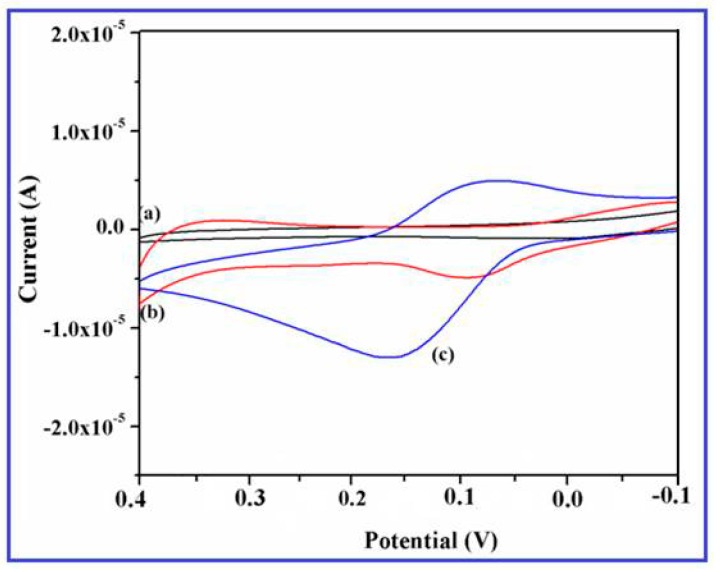
Cyclic voltammograms of (**a**) unmodified glassy carbon electrode (GCE), (**b**) CuO nanowires/GCE in PBS (pH 7.4), and (**c**) CuO nanowire/GCE at 0.2 mM DA dissolved in 0.1 M phosphate buffer (pH 7.4) solution recorded at a scan rate 50 mV/s.

To examine the electrocatalytic response of CuO nanowires synthesized using SMFE, it has been exploited for their intriguing properties on the electrochemical sensing of DA on CuO nanowires/GCE. The cyclic voltammograms of 0.2 mM DA on the bare GCE and the CuO nanowires/GCE in the potential range of −0.2 V to +0.4 V at a scan rate 50 mV/s are illustrated in the Figure 7. No characteristic redox behavior was observed for the bare GCE (Figure 7a), whereas the CuO nanowires/GCE displayed a small background current (Figure 7b) in the PBS (0.1 M).

This tremendous redox behavior of DA revealed the excellent electrocatalytic activity and large surface to volume ratio of the CuO nanowires that were coated on the electrode surface. However, after the addition of 0.2 mM DA, a pair of well-defined redox peaks is observed at an anodic oxidation potential of 0.18 V and cathiodic reduction potential of 0.13 V with a dramatic increment of current response (Figure 7c). These results specified that the SMFE assisted CuO nanowires significantly enhances the electrochemical property of the modified electrode, which may be ascribed to the higher surface energy, large available surface area, and improved electron transfer reaction of the CuO nanowires on the GC electrode [46]. Moreover, it also demonstrated that the effective electroactive surface area of the electrode was considerably increased by employing the CuO nanowires as a electrode material, it may be attributed to the anticipated involvement of surface species (Cu(II)/Cu(III) ions) during the electrocatalytic oxidation towards DA.

### 3.9. Redox Behavior of Dopamine

**Scheme 2:**
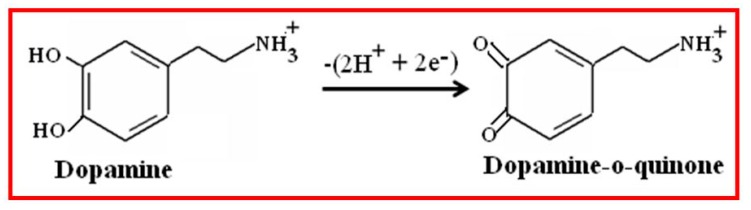
Electrochemical Oxidation Mechanism of Dopamine on CuO nanowires/GCE.

The presence of negatively charged free surface hydroxyl groups of CuO nanowires have been confirmed through FTIR study. These surface hydroxyl (–OH) groups preferentially undergoes chemical bonding with the positively charged amine (–NH_2_) groups of dopamine at the physiological pH 7.4, which could form hydrogen bond and facilitate the electron transfer reaction of DA. Also, the stimulated conducting activity of CuO nanowires on the specified potential greatly increased the magnitude of current response than the bare GCE. When an appropriate potential is applied to the CuO nanowires/GCE, it demonstrates that the electrochemical oxidation reaction of DA occurs by exchanging the two electron and two protons, and thus the dopamine-o-quinone was formed [47]. According to the above conversation and the well known fact, the electrocatalytic oxidation behavior of DA on CuO nanowires/GCE can be elucidated by Scheme 2.

### 3.10. Influence of the Scan Rate on the Detection of Dopamine

**Figure 8:**
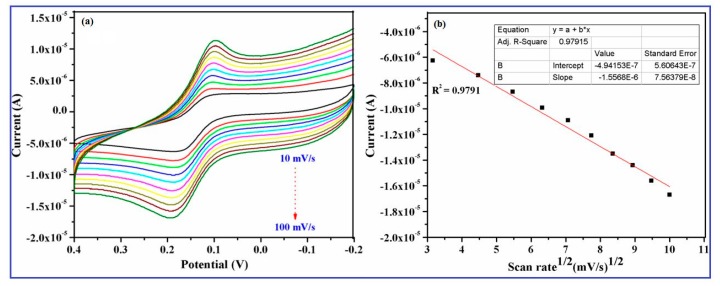
Cyclic voltammetric responses of CuO nanowires/GCE in the presence of 0.2 mM DA in 0.1 M phosphate buffer (pH 7.4) (**a**) at different scan rates (10 mV/s to 100 mV/s) and (**b**) calibration curve plotted versus square root of the scan rate and oxidation current.

The electrochemical response of the CuO nanowire/GCE in 0.2 mM dopamine with the effect of the scan rate is shown in the Figure 8a. The oxidation and reduction peak currents simultaneously increased with the increasing scan rate from 10 mV/s to 100 mV/s and its corresponding calibration plots of the oxidation peak current vs. square root of scan rate are given in the Figure 8b, which illustrates the linear toward various scan rates on the modified electrode.

It can also be seen that the oxidation and reduction peak potentials shifted toward more positive and negative direction, respectively. The correlation coefficient corresponding to the anodic peak current was 0.9791, which indicates that the diffusion processes control the overall kinetics [48]. In the determined range of scan rate, the oxidation peak current linearly plotted to the square root of the scan rate and the peak separation became larger with the increase of scan rate. It can also be seen that the potential gap between oxidation and reduction peak current did not significantly shift, exhibiting the enhanced electrochemical activity was performed on the CuO nanowires/GCE.

### 3.11. Effect of Dopamine Concentration on the CuO Nanowires/GCE

**Figure 9:**
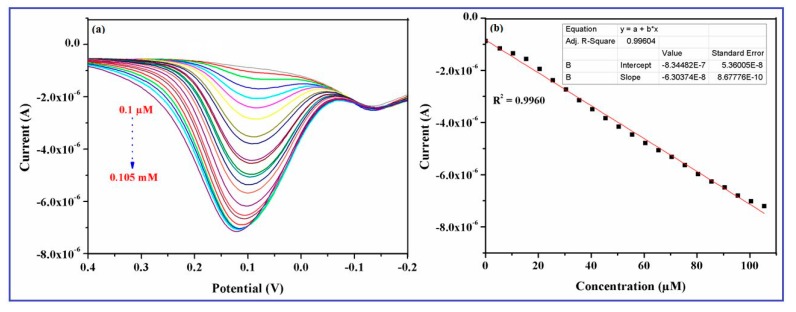
Differential pulse voltammetry (DPV) responses obtained for various concentrations of DA (0.1 µM to 0.105 mM) in 0.1 M phosphate buffer (pH 7.4) at a fixed scan rate 50 mV/s (**a**) CuO nanowires/GCE and (**b**) calibration curve plotted versus oxidation current and the molar concentration of dopamine (DA).

The DPV method can be used to determine the electrocatalytic activity of DA because of the high current sensitivity and resolution of this technique. An excellent electrochemical performance of the green synthesized CuO nanowires for the determination of DA was also demonstrated through the DPV measurements in 0.1 M PBS with various concentrations (0.1 μM to 0.105 mM) of DA, which are shown in the Figure 9a.

The result indicated that anodic oxidation current successively increased with the increasing concentration of DA in the PBS, which proves the electrocatalytic response of the modified electrode for DA sensing is highly concentration dependent [49]. This type of immediate response on the electrode implies that the material (CuO nanowires) efficiently provides the well-defined voltammetric signals by promoting the redox behavior of DA. Figure 9b illustrates the relationship between the different concentrations of DA and the corresponding anodic peak currents, with a correlation coefficient of 0.9960. The obtained result indicates that the detection of very low concentrations of DA is possible on the CuO nanowires/GCE and the proposed biosensor showed a wide linear range of DA from 0.1 μM to 0.105 mM, with an acceptable sensitivity and limit of detection of 0.1 μM. The oxidation current response became saturated at a concentration of 0.10 mM, which may be accredited to the availability of a large number of nanowires with higher electro-active surface area, promotes electron transfer reaction of DA. In addition, the sensing performance of the newly designed biosensor was also compared for the detection of DA with the various nanostructured electrode materials. The linear range, detection limits (LOD), optimal pH of the electrolyte, and techniques used for the other electrode materials are summarized in Table 1, and the CuO nanowire/GCE showed better electrochemical performance than the previously reported DA biosensors.

### 3.12. Anti-Interference/Selectivity Study on the CuO Nanowires/GCE

**Figure 10:**
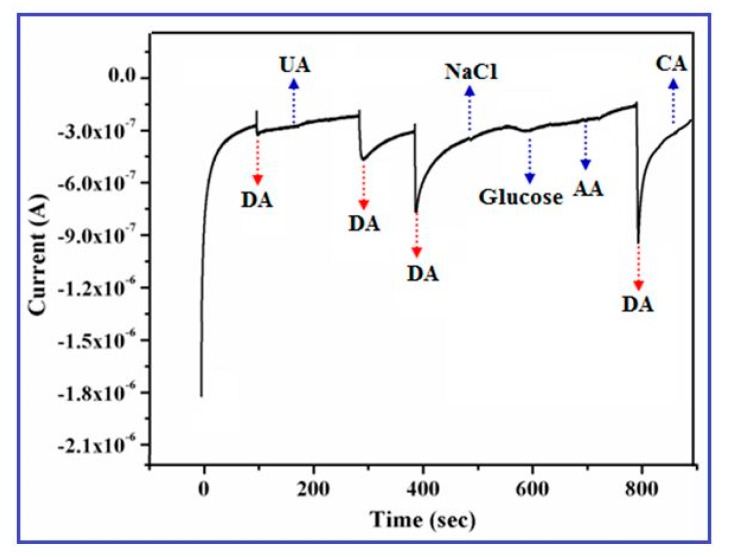
Interference study of the CuO nanowires/GCE recorded in 0.1 M phosphate buffer (pH 7.4) at a fixed potential of 0.15 V with the existence of UA, NaCl, AA, glucose and CA.

In order to assess the selectivity and response of the CuO nanowire/GCE in the presented DA sensor, examination of the electrocatalytic properties with the influence of common interfering biomolecules/species, such as AA, UA, CA, NaCl, and glucose was evaluated by using chronoamperometry at a detection potential of +0.15 V. Figure 10 displays the amperometric response to the consecutive additions of 0.1 mM of DA, glucose, AA, CA, UA, and NaCl in 0.1 M PBS (pH 7.4) solution.

The outcome of the experimental result represents that the above interfering species did not show any remarkable change in the oxidation current response of DA that explored the outstanding selectivity of the proposed sensor. Further, the anodic current response occurred by DA is extremely higher than the other interfering species (AA, glucose, CA, NaCl and UA), which implies good selectivity and sensitivity of CuO nanowires/GCE towards the quantitative analysis of DA even in the presence of general physiological interfering biomolecules.

### 3.13. Selectivity Study on the CuO Nanowires/GCEusing CV and DPV

**Figure 11:**
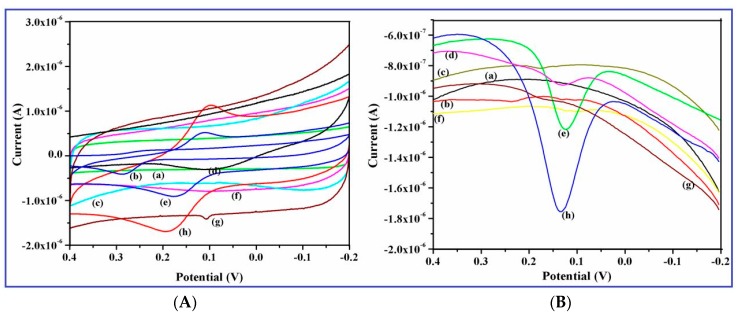
Interference studies on CuO nanowires/GCE recorded in 0.1 M phosphate buffer (pH 7.4) in the absence and presence of (a) blank (b) 100 µM UA (c) 100 µM CA (d) 100 µM AA (e) 100 µM DA (f) 100 µM Nacl (g) 100 µM glucose and (h) mixture of 100 µM of each species (**A**) cyclic voltammetry (CV) curves and (**B**) DPV curves, respectively.

Interference study was further examined by CV and DPV using possible interferents, such as UA, AA, CA, Glucose, and NaCl for the selective determination of DA. It can be seen that both the CV and DPV curves exhibited the similar electrochemical responses on the modified GCE. Figure 11A,B represents the CV and DPV curves of individual UA, AA, DA, CA, Glucose, NaCl and their mixture at the CuO nanowires/GCE in pH 7.0 PBS, it can be noted that a pair of redox peak is appeared for DA and the common interfering species, like UA and AA, showed small oxidation current, whereas other species does not observe any redox behavior on the modified GCE. On the other hand, the electrochemical reaction of the mixture species exhibited only clear redox behavior of DA and hinders the oxidation process of UA and DA. Therefore, we confirmed that it can clearly detect the remarkable enhancement of DA redox behavior at CuO nanowires/GCE with the presence of possible concomitants and it promotes the selective determination of DA.

### 3.14. Stability/Reproducibility of the CuO Nanowires/GCE

**Figure 12:**
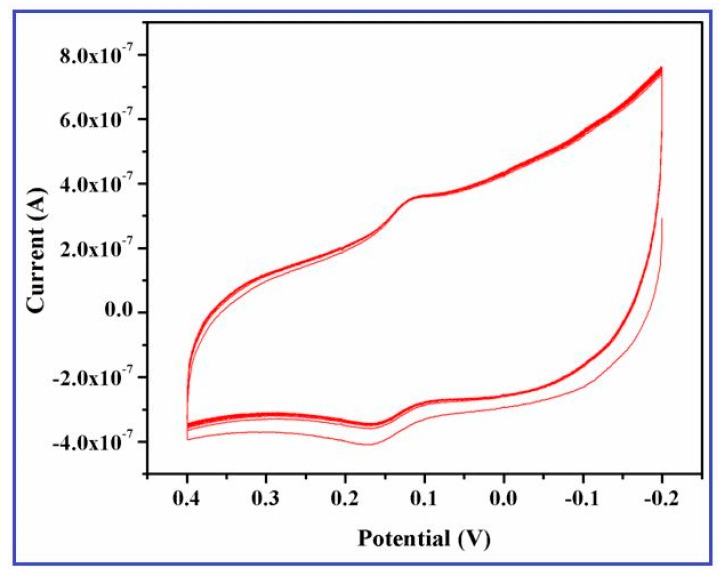
Fifty repeated CV scans of the CuO nanowires/GCE in 0.2 mM DA at a scan rate 50 mV/s.

Figure 12 illustrates the reproducibility and stability of the CuO nanowires/GCE towards 0.2 mM DA for the estimation of the sensing performance and the stability was tested using the electrode for 50 multiple CV cycles.

Only a 5.8% decrease of redox peak current was observed, even after 50 cycles, indicating the good stability of the electrode. In the reproducibility tests, six independently fabricated CuO nanowires modified electrodes were investigated and the electrochemical performance of each modified electrode was measured in a 0.2 mM DA solution. The estimated relative standard deviation for the oxidation of DA was calculated to be 2.36%. The observed results obviously displayed the adequate reproducibility of the newly designed electrode material (CuO/nanowires) toward the determination of DA.

## 4. Conclusions

We have developed an efficient electrochemical biosensor using CuO nanowires and tested towards the selective and sensitive determination of DA. The CuO nanowires, like morphology, were developed through a facile green synthetic protocol employing *Sapindus mukorossi* fruit extract as a biosurfactant. The structural, crystalline, and morphological characteristics of the CuO nanowires were investigated using XRD, XPS, FTIR, FE-SEM, and HR-TEM analyses. This green synthesis method is a versatile, large-scale, cost-effective, and spontaneous production method for CuO nanostructures for energy and biological applications. Here, the proposed CuO-nanowire-modified sensor showed high selectivity and sensitivity, a low detection limit, and a relatively wide dynamic range, demonstrating its potential for the development of an eco-friendly platform for the detection of dopamine or related neurochemicals. Furthermore, the valid response to DA obtained in this present work indicates the promise of the CuO nanowires for the detection of other biological molecules, such as nucleic acids, proteins, and enzymes. This new sensor opens opportunities for the fast, simple, and selective detection of DA and it provides a promising platform for biosensor applications.

## Figures and Tables

**Table 1:** Comparison of some dopamine sensors based on different electrode materials.

Electrode Material	Technique	LOD	Linear Range	Electrolyte	Ref.
Au-graphene	DPV	1.86 mM	5–1000 mM	pH 6.0	[50]
Graphene	DPV	2.64 mM	4–100 mM	pH 7.0	[51]
3D-GN@WO_3_ nanowire	CA	238 µM	10–150 mM	pH 6.0	[52]
Au-Cu_2_O/rGO	DPV	3.9 µM	10–90 µM	pH 7.0	[53]
GO	DPV	0.27 mM	1–15 mM	pH 5.0	[54]
Au/RGO/GCE	DPV	1.40 μM	6.8–41 μM	pH 7.0	[55]
NiO-CuO/GR/GCE	SWV	0.167 μM	0.5–20 μM	pH 8.0	[56]
RGO-Pd-NPs	LSV	0.23 μM	1–150 μM	pH 7.0	[57]
Rod shaped CuO nanoparticles/MCPE	DPV	0.18 μM	0.3–1.4 μM	pH 6.0	[58]
Ag/RGO	LSV	5.4 µM	10–800 µM	pH 6.0	[59]
CuO/MWNTs/Nafion/GCE	DPV	0.4 µM	1.0–80 µM	pH 6.0	[60]
ZnO/MWNTs/GCE	CV	3 µM	3—200 µM	pH 7.0	[61]
CuO nano-rice/GCE	DPV	0.42 µM	1–150 µM	pH 7.0	[62]
GR-SnO_2_/CILE	DPV	0.5 µM	5–500 µM	pH 6.0	[63]
N-doped graphene	DPV	0.25 µM	0.5–170 µM	pH 6.0	[64]
GR-CS/GCE	DPV	5 µM	15–175 µM	pH 7.0	[65]
CuO nanowire/GCE	DPV	0.1 µM	0.1–105 µM	pH 7.4	This work
